# Career perspective: Paolo Cerretelli

**DOI:** 10.1186/2046-7648-2-13

**Published:** 2013-04-25

**Authors:** Paolo Cerretelli

**Affiliations:** 1 Istituto di Bioimmagini e Fisiologia Molecolare del Consiglio Nazionale delle Ricerche (C.N.R.), via Fratelli Cervi, 93, Segrate, Milan 20090, Italy

**Keywords:** High altitude, Acute and chronic hypoxia, Chemoreflexogenic ventilatory drive, *V*′O_2_max, Anaerobic glycolysis, Cardiac output, Skeletal muscle, Muscle proteomics, Tibetans vs. Caucasians

## Abstract

This article is an autobiographical account of my career as a human physiologist. I have spent 55 years traversing mountains, continents, seas, and skies, carrying out research in the laboratories of several international institutions as well as in the field. My scientific roots, approach to the mountains and altitude populations, both in Europe and in Asia, together with an account of my experimental studies at altitude, including extreme conditions, shall be presented together with pertinent occasional reflections of a personal nature.

## Introduction

The Editors' kind invitation to recall my research activity in ‘extreme’ conditions, particularly at altitude, over the last five decades reached me a few months before my eightieth birthday (born 21 October 1932 in Milano, Italy) in the course of the third (and the last) stage of my retirement itinerary. Indeed, I am still active as an associate scientist at the Italian Research Council Institute of Bioimaging and Molecular Physiology in Milano.

I retired officially the first time in 1997, after serving 20 years as a professor of physiology at the Geneva University Medical School (Switzerland) and was made honorary professor. The second pensionable age was attained in Milano in 2007, after a further decade spent at the medical school of the local state university: this deferred period concluded my initial 20 years of service (1958–1978) during which I had progressed, under the guidance of my mentor Rodolfo Margaria, from an instructor in Physiology to a full professor (1969) and chairman (1972). My activity in Milano and Geneva was interspersed with several sojourns abroad:

1. In *Pakistan*, for 4 months in 1959, as a member (M.D.) of a mountaineering expedition in the Karakoram (Kanjut-Sar, 7,760 m),

2. In *Germany* (Göttingen) at the Max Planck Institut für Experimentelle Medizin with Johannes Piiper (for 6 months in 1962, with fellowships from the European Community (OECD) and the Max Planck Society) and later, over several periods, being involved together with Pietro Enrico di Prampero in collaborative Milano/Göttingen projects on metabolism, respiration, and circulation in isolated dog muscle and exercising animals,

3. In the *USA* at the Physiology Department of the State University of New York at Buffalo (from 1963 to 1965 and from 1976 to 1978, initially as a recipient of a NATO fellowship and a visiting assistant professorship and later as a visiting research professor of physiology with Hermann Rahn, Leon Farhi, Donald Rennie, and David Pendergast working in man on respiratory gas exchange at rest and exercise, on the development of bloodless methods for the measurement of cardiac output and, particularly, on the assessment of its rate of adjustment at the onset of exercise; in ‘extreme’ divers, analyzing with David Pendergast, Massimo Ferrigno, Guido Ferretti, and Claes Lundgren gas exchange and cardiovascular functions in the open sea and in a ‘wet’ hyperbaric chamber; and in collaboration with Claudio Marconi and David Pendegast, on the readjustment rate of muscle microcirculation in the course of exercise, both in man (from ^133^Xe clearance) and in the dog (by radioactive microsphere trapping),

4. In *Nepal*, while based in Milano (1973), as a deputy leader, responsible for physiological research, of the first successful Italian Mt. Everest Expedition (eight summiteers, of whom five were Italians (one of them just 20 years old) and three were Sherpas (one of them 19 years old)). Later, from Geneva, I had the opportunity to coordinate with Pietro Enrico di Prampero, Hans Hoppeler, Hans Howald, and Guido Ferretti a multidisciplinary study on the effects of altitude on muscle structure, ultrastructure, and function (the first after prolonged exposure to extreme altitudes) in members of the 1981 Swiss expedition to Mt. Lhotse Shar (Nepal, 8,398 m), and of the 1986 Swiss expedition to Mt. Everest [1] and to carry out, with Oswald Oelz, an investigation on the physiological profile of world-class high-altitude climbers [2]. In the 1990s, I worked in the Khumbu Valley at Namche Bazar and Kumjung and, on repeated occasions, at the Pyramid laboratory, the permanent structure built at Lobuche (5,050 m) by professor Ardito Desio who was a famous Himalayan geologist, on muscle aerobic and anaerobic metabolism in man in the course of altitude acclimatization with Claudio Marconi, Bruno Grassi, Mauro Marzorati, Bengt Kayser, Marco Narici, Michael Meyer, and several younger colleagues (see paragraph on ‘Research at the Pyramid’) as well as on blood oxygen affinity in acclimatized Caucasians and altitude Sherpas with Michele Samaja and Arsenio Veicsteinas [3].

Upon return to Milano, I organized with Claudio Marconi and Cecilia Gelfi at the Tibetan Refugee Reception Center in Kathmandu (2002) the first investigation on muscle proteomics in altitude Sherpas. More recently, I was involved in studies of muscle molecular adaptation in acute, sub-acute, and chronic hypoxia occurring in man after sojourns in the Alps (Margherita Hut, Italy, 4,559 m) with Cecilia Gelfi and Carsten Lundby and at Mt. Everest within the Caudwell Xtreme Everest (CXE) 2007 Research Expedition with Mike Grocott, Denny Levett, and Cecilia Gelfi.

Time runs incredibly fast, the more so when growing older! When the Editors of this journal contacted me, I hesitated for a couple of weeks before accepting the challenge to go through the most significant stages of my career. However, in retrospect, this has offered me the opportunity to revive memoirs of innumerable events, people, and places. I must admit that being ‘forced’ to recall several ‘flashbacks’ of my life has often induced deep nostalgia, but fortunately, there has never been time for sadness: for this, thanks to the privilege of being surrounded by many sympathetic younger co-workers who encouraged me to take up the invitation, showing also some unexpected interest for many aspects of my rather unusual career. A handshake to everyone!

## My scientific roots

Despite earlier occasional descriptions of mountain sickness and reduced exercise capacity at altitude correctly attributed to lack of oxygen, the recognized father of modern high-altitude physiology and medicine has been Paul Bert (1833–1886). His work, mainly carried out in the famous laboratory at the Sorbonne in Paris, is described in the monumental book *La Pression Barométrique*[4]. Bert was the first or, more precisely, the second after his patron Denis Jourdanet to attribute the deleterious effects of low barometric pressure, both in hypobaric chambers and at high altitude, to reduced oxygen in blood or ‘barometric anoxemia.’

As is well known, the initial, significant progress in the studies of human respiratory, cardiovascular, and exercise physiology and physiopathology at altitude took place mainly in the last two decades of the nineteenth and in the first of the twentieth century when facilities for experimental studies could be installed in the Alps, on Mont Blanc (1890) by J. Vallot (observatoire Vallot, 4,350 m) and, particularly, in 1893 on Monte Rosa (at the Capanna Margherita, 4,559 m) by the Italian Alpine Club on the initiative and under the direct supervision of the great scientist Angelo Mosso (1846–1910) who was a professor of physiology at the University of Torino. Extensive field studies were carried out around the turn of the nineteenth century essentially by Mosso himself, as reported in his invaluable contribution *L'Uomo sulle Alpi*[5], and by N. Zuntz, A. Loewy, F. Müller, and W. Caspari published in their famous book *Höhenklima und Bergwanderungen in ihrer Wirkung auf den Menschen*[6]. Shortly afterwards, altitude research was extended to other remote locations such as the Alta Vista Hut (3,350 m on Tenerife in the Canary Islands, expedition led by Zuntz with the participation of J. Barcroft in 1910) and at the Pikes Peak (4,300 m) in the USA (Anglo-American Expedition (1911) with J.S. Haldane, E.C. Schneider, Y. Henderson, C.G. Douglas, and M. P. FitzGerald).

Angelo Mosso, the forerunner and an important figure in high-altitude research, was trained in zoology at the University of Torino and obtained his doctorate in medicine in 1870 with a thesis on the growth of bones. After doing military service as medical officer in Southern Italy, he spent a short period at the University of Florence at the Physiology Laboratory of M. Schiff whence, in 1873, he moved to the laboratory of K. Ludwig in Leipzig (Germany). Here, he became adept at constructing and handling the kymograph, an instrument just invented by his host and which proved to be essential for recording a number of physiologic mechanical variables such as those generated by his famous ergograph, by his sphygmograph (the prototype of the well-known FINAPRES), and by the Marey pneumograph. Subsequently, he visited in France the laboratories of C. Bernard, L. Renvier, and E. Marey before returning to Torino (1875). He was appointed professor of physiology in 1879 when his mentor J. Moleschott moved to Rome as chairman of physiology at La Sapienza. Mosso founded the first laboratory of cardiovascular, respiratory, muscle, and exercise physiology that became a mecca for many young Italian physiologists and was also visited by well-known physiologists from abroad. He was the forerunner of high-altitude physiology and medicine in Italy, the scientist who, besides the laboratory at the Capanna Margherita, promoted in 1907 the construction of the Istituto Scientifico Angelo Mosso at the Col d'Olen (2,900 m). The latter was the result of a joint venture supported financially by the King and Queen of Italy and the Italian government, with substantial contributions by the scientific communities of all alpine countries and various public and private sponsors from the UK, Belgium, Holland, and the Washington Academy of Sciences. The Institute was named after Mosso following the proposal of the general assembly of the 7th International Physiology Congress at Heidelberg (1907).

Mosso was keen on human research. He used his brother (medical doctor and, eventually, professor of pharmacology at the University of Genova), his faithful technician G. Mondo, and a number of young soldiers, students, and some patients as subjects for his experiments. These were carried out in the laboratory of Torino (ergographic recordings, measurements of gas exchange, and tracings of brain pulsations in patients exposed to severe hypoxia in a small hypobaric chamber) and at the Capanna Margherita (recordings of periodic breathing, probably the first in the literature, and observations that acute hypoxia, despite blood O_2_ desaturation, does not affect the maximum mechanical performance by small muscle masses, such as those of the forearm (handgrip) and the arms). The latter results could be explained only recently by a compensatory increase of regional blood flow when exercising in hypoxia. Moreover, to my knowledge, Mosso was the first to propose, on the basis of his observations of increased and persistent luminescence of fireflies exposed to deep hypoxia, the existence of chemical energy sources in the cell that are independent of oxygen supply, i.e., the possibility of incurring an O_2_ debt, likely alactacid. Despite his scientific rigour, Mosso is also known for an erroneous statement (even though based on correct findings of extremely low concentrations of carbon dioxide in expired air during exposures of a co-worker, G. Mondo, to simulated altitudes up to 8,800 m in his hypobaric chamber) that the deleterious effects of high altitude depended on low CO_2_ (‘acapnia’) rather than O_2_ (‘hypoxia’) pressure levels in the blood. His conclusion was supported by the observation of the beneficial effects of CO_2_ administration to a monkey exposed to deep hypoxia in a hypobaric chamber. Mosso's observations were correct, but the explanation of the cause was challenged by Loewy (1895) and later by Barcroft (1910) on the basis of measurements of blood oxygen pressure. About 50 years later, the solution of the alveolar air equation for hypoxia provided the answer: Indeed, hypocapnia is beneficial for it induces hyperventilation, thereby leading to relative increases of alveolar O_2_ partial pressure (*P*_A_O_2_) for any given *P*_I_O_2_ level.

The first generation of Mosso's followers was that of his disciple Amedeo Herlitzka and the collaborators of the latter among whom I would like to mention Alberto Aggazzotti, the first director of the Institute at Col d'Olen until 1923 (when he was appointed as professor of physiology at the University of Modena), not only for his pioneering studies on compensated acidosis at altitude but also for the construction of the first manometric micro-O_2_ and micro-CO_2_ analyzer (the prototype of the future Scholander apparatus) for studying gas exchange in the chick embryo as reported by Rahn [7].

Among the members of the second generation, my future boss Rodolfo Margarìa was undoubtedly the most distinguished respiration and exercise physiologist ascending to a position of pre-eminence in Europe and the USA. Born in Chatillon which is just below Mt. Cervino (The Matterhorn), he was educated at the University of Torino where, in 1924, he obtained a medical degree. After working for a few years with Herlitzka, he moved to London (1930–1931) working on a Rockefeller grant at the University College London with A.V. Hill, of whom he was a personal guest, at Cambridge with J. Barcroft (1931), in the USA at Harvard with L.J. Henderson at the Fatigue Laboratory (1932), in New York with D.D. Van Slyke, and in 1934, as a fellow of the A. Volta Foundation in Heidelberg (Germany) with O. Meyerhof, the biochemist who shared the Nobel Prize in 1923 with A.V. Hill. He then became a professor of physiology at the universities of Ferrara, Parma, Pavia and, finally, Milano (1938–1972). Margaria had broad biological interests in the biochemistry of hemoglobin, in exercise bioenergetics, and in environmental physiology with a special interest for high-altitude and, in his later years together with Giovanni Cavagna, for mechanics of locomotion particularly in microgravity.

## My approach to physiology

As a schoolboy, following the example of an uncle who was a doctor and not of my father who was an industrial research engineer who matured professionally between the two world wars at General Electric in the USA, I decided to undertake medical studies (1949/1950) at the University of Milano. My first contact with physiology was towards the end of 1952 when I began my internship at the Institute of Physiology and Biochemistry of the Medical School at the University of Milano directed by professor Margaria. Within a group of advanced students, I had the opportunity to participate in some experimental activity and attend the weekly seminars on various aspects of the research work going on in the laboratory, such as the regulation of hemoglobin O_2_ affinity by hydrogen ions and CO_2_, some features of breathing mechanics, and the technique for the measurement of the time lag of spinal reflexes in man. After taking exams in biochemistry and physiology, I moved to the Department of Internal Medicine assigned as an intern to the Section of Rheumatology where I spent over 3 years (1953/1956) learning medical pathology under the guidance of the staff. From the outset, I could enjoy the spectacular progress in the treatment of rheumatic diseases, particularly rheumatoid arthritis just as the new antirheumatic drugs, such as butazolidin and steroids, became available. Meanwhile, I could work at my experimental thesis to obtain a medical grade on the *Output of urinary glycoproteins in rheumatic, neoplastic and inflammatory diseases*. When defending my thesis (July 1956), I was informed that professor Margaria was looking for instructors in physiology, and I went to visit him. He recognized me as a former intern at his laboratory and offered me a part-time temporary job while I waited for admission (national service) to the school of medical officers in Florence (6 months). Subsequently, I worked for 1 year at the military hospital in Milano and was able to spend some time also at the Department of Physiology of the university. Soon after discharge (March 1958), I was fortunate enough to obtain a position of assistant professor in physiology. After studying the most important papers by Margaria with D.B. Dill on the origin of the O_2_ debt from the Fatigue Laboratory in Harvard, interacting with young colleagues who I met in the laboratory (including E. Agostoni and G. Milic-Emili) and learning to use the available equipment, I took up the option to work with Margaria in exercise and environmental physiology. My choice was also promoted by the increasing number of requests for collaboration from sports institutions and athletes in light of the approaching Olympic Games (Rome, 1960) and by some organizers of mountaineering expeditions who often required the advice of Margaria, for example, during the preparation of the first successful climb of K2 by the expedition of Desio in 1954.

## My approach to the mountains

**Figure 1:**
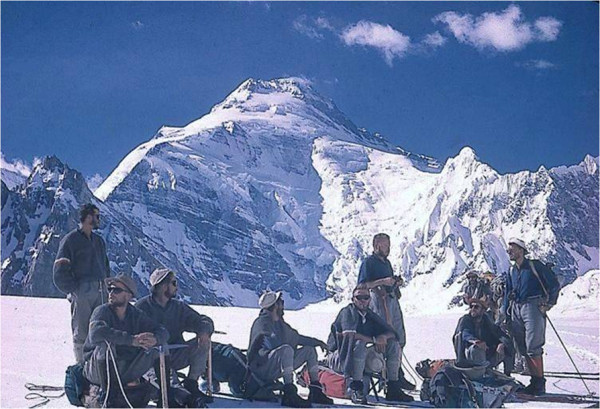
**The Kanjut-Sar (7,760 m).** View from the entrance to the Khani Basa glacier.

The quite unexpected invitation to participate in a mountaineering adventure occurred in late 1958 when I was involved in the functional evaluation of the candidate members of the expedition to Mt. Kanjut-Sar (7,760 m, Karakoram, Pakistan; Figure 1).

The organizer and the leader of the expedition was a young, wealthy entrepreneur fond of mountains and, for several years, a supporter of a group of professional alpine guides of Valtournenche/Cervinia, the resort at the foot of the Mt. Cervino (the Matterhorn), and the leader of previous expeditions within the Alps and to the Andes. Upon invitation of the leader, with the encouragement of Margaria and the approval of the chancellor of the university, I was engaged as the doctor (also with some administrative tasks) for a group of 11 (the leader, eight guides, a camera man, and an expert in logistics, as well as for several hundred Hunza porters mainly from the two semi-autonomous emirates (under Pakistani rule) of Hunza (Baltit) and Nagar). At the time of departure, my experience with the mountains was that of a tourist, with the exception of a preliminary climb of the Breithorn with a group of guides for testing oxygen masks and likely…my physical fitness. My only request was the availability of six porters (one of whom, previously, was the batman of a doctor in the local army) for carrying my research equipment and medicines. As suggested to me by a veteran Himalayan explorer, the latter proved to be really essential for establishing friendly contacts with natives emerging from the middle of nowhere along the Hispar Glacier looking for any kind of medical treatment, from surgery for the removal of a goiter (!) to a shot of vitamin C. By the way, the most spectacular results of my interventions were from the use of eye drops containing hydrocortisone and chloramphenicol on trachoma which made me a wizard!

**Figure 2:**
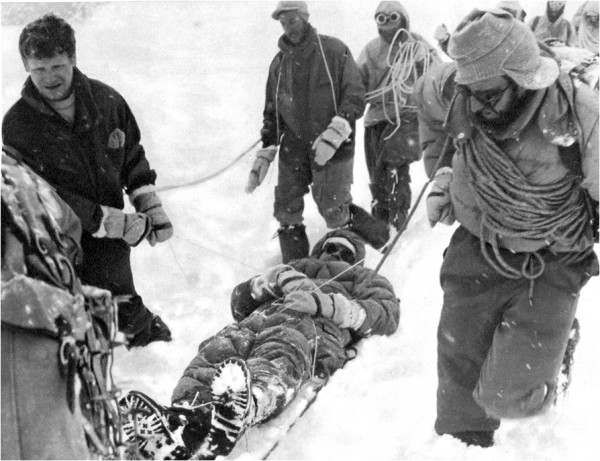
**Assisting the leader of the expedition on the way back to the base camp. **Courtesy of G. Monzino, from *Kanjut-Sar*, edited by A. Martello, Milano (1961).

My participation in the Kanjut-Sar Expedition had a heavy impact to me due to its long duration (over 4 months, the lengthy approach march lasting over 20 days), for an almost total lack of contact with the civilized world or communication within the group, with most members speaking among themselves in their incomprehensible ‘patois,’ for the dreariness and the danger of the environment, the lack of drinking water, fruit and vegetables, and soon, the repulsion for canned food. The base camp was set at the top of the Khani Basa, a tributary of the Hispar Glacier, at about 4,960 m above the crevasses. According to the literature, no one had ever been there before us. By and large, the situation was that of a total confinement further worsened by the sudden death of an altitude porter while recovering from a fall after days of assisted ventilation in a tent with an oxygen-enriched gas mixture. From bad to worse, the conditions were complicated with the leader of the expedition falling seriously ill. He laid sick, practically unconscious for 2 weeks in my tent after having been rescued from an advanced camp hit by a snow storm (Figure 2).

I had been asked by the leader, in the case of his inability to do so, to take the responsibility for the expedition together with the head of the guides and the Pakistani liaison officer, and so I did. Shortly afterwards, therefore, the climbing program continued, the summit of the mountain was reached in ‘a solo’ climb by Camillo Pellissier, practically without supplemental oxygen, and I could honor my promise (repeatedly solicited upon departure) to the mother of the leader of the expedition to bring him back safe. By the way, my reward, negotiated by exchange of telegrams before returning from Karachi, was a large bowl of tomatoes served during a magnificent dinner party at the residence of the lady, the Convento dei Cappuccini on the Canal Grande in Venice!

With hindsight, compared with the Kanjut-Sar experience, all expeditions in which I took part later, three of them to Greenland, several ‘light’ ones on the occasion of field studies carried out in Kenya, in the Khumbu Valley of Nepal, and in Chilean Patagonia and even that to Mt. Everest, appear to me now as working vacations, thanks to perfect planning and organization, availability of laboratory facilities, equipment and power supplies for heating, telecommunications, and for all kinds of comfort food and beverages, including champagne! Notwithstanding, several among the participants in the various events felt often exhausted, developed symptoms of failure, and had to quit ahead of time. This is the proof that what is really essential for explorers and altitude climbers, particularly those involved in long lasting displacements or scientific projects, is motivation and a sound psychological structure as always claimed, among others, by Reinhold Messner.

## My contribution to altitude research

### The Kanjut-Sar Expedition

**Figure 3:**
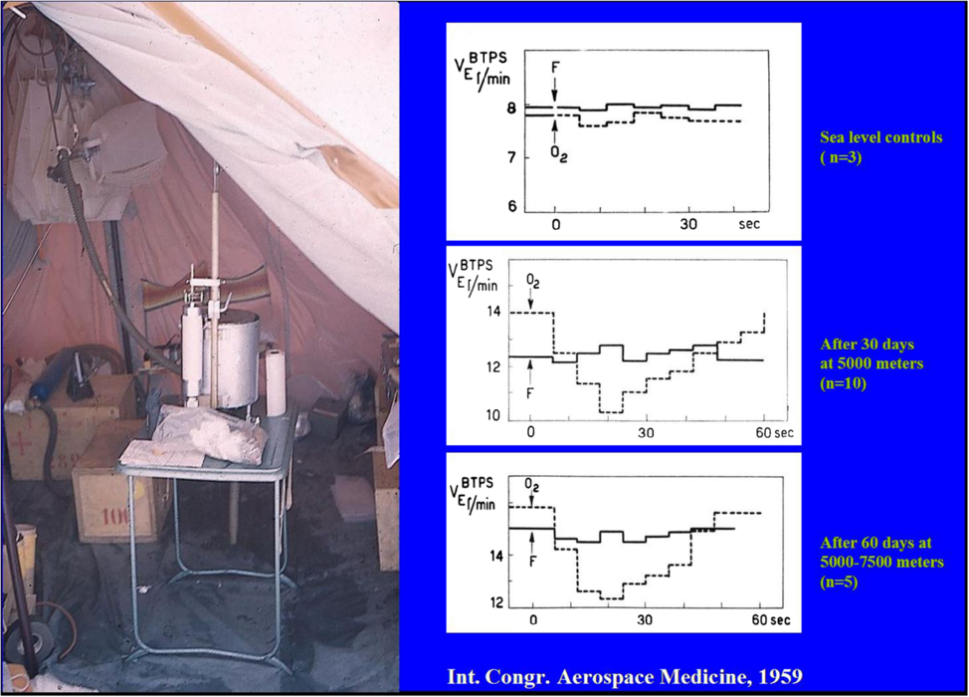
**Setup for the measurement of pulmonary ventilation breath by breath. **Following administration of single tidal volumes of oxygen or ambient air.

For the reasons given above, carrying out some experimental work at the Kanjut-Sar base camp (4,960 m; BP = 410 mmHg) became for me a categorical imperative. Four series of experiments were performed:

1. In the first, vital capacity was determined in 11 subjects after 60 days of exposure between 5,000 and 7,500 m. The results indicated a reduction by 12.5%. Parenthetically, maximal exercise ventilation decreased (*n* = 5) by 21.8%.

2. In the second, *P*_A_CO_2_ and *P*_A_O_2_ were determined at rest in seven subjects after a 60-day sojourn between 5,000 and 7,500 m. The data were compatible with the line appearing in the Rahn-Otis diagram for 5,500 m [8].

3. Maximum oxygen consumption (*V*′O_2_max) was found in five subjects to be decreased by over 50% at 4,960 m after a 60-day sojourn between 5,000 and 7,500 m, i.e., significantly more than in equivalent acute hypoxia despite a 30% increase in hemoglobin concentration [Hb] [9].

4. A persistent chemoreflexogenic drive assessed by chemical denervation of the carotid bodies (obtained by administration of tidal volumes of pure oxygen) was observed throughout 60 days of exposure to altitudes of 5,000–7,500 m (Figure 3) [10].

### The Mount Everest Expedition and derived studies

This very large 64-man expedition was organized by a private group with the very consistent support of the Italian Defense Ministry that provided a large number of military participants, most of them non-mountaineers, the logistics (planes for transporting men and equipment from and to Italy), telecommunications as far as the base camp, electric power supply, and two helicopters for transporting equipment along the Khumbu Valley and eventually to the base camp (5,350-m altitude). By the way, helicopter takeoff had never been attempted before above 4,500 m and was not officially permitted by the factory: Throughout the approach to the base camp, pilots and engineers made several attempts to raise the takeoff ceiling mainly for technical research purposes. One of the helicopters adapted for extreme takeoff and landing on ice was lost without casualties when trying to land at the advanced base camp (6,300 m) to evacuate a high-altitude Sherpa who had developed acute pulmonary edema (the only case in my personal experience). The second helicopter was able to rescue the patient and the three members of the crew of the crashed one and, on the way back, picked me up at the base camp to take care of the patient, who was unconscious, during the transfer to Kathmandu. After inflight standard treatment, upon landing, he was able to walk, and within a few days of hospitalization, he fully recovered.

**Figure 4:**
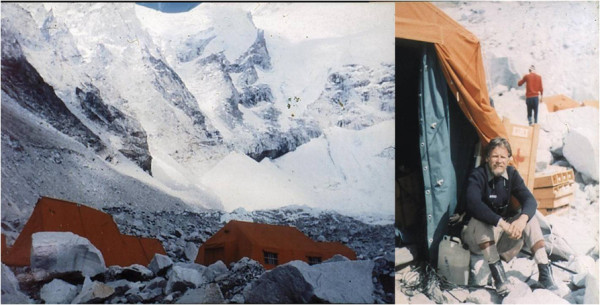
**The laboratory tent at the Mt. Everest base camp. **With the author at the entrance.

**Figure 5:**
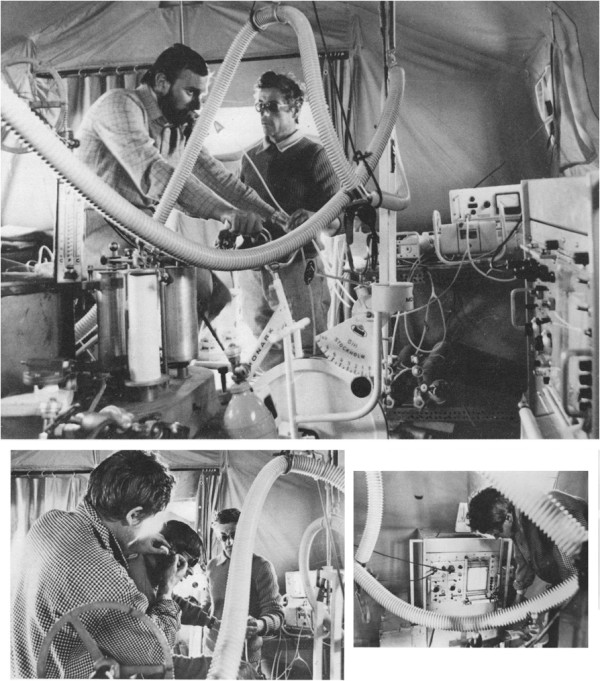
**Exercise laboratory at the base camp. **A bicycle ergometer, spirometers, gas analyzers, and electronic recorders are visible.

**Figure 6:**
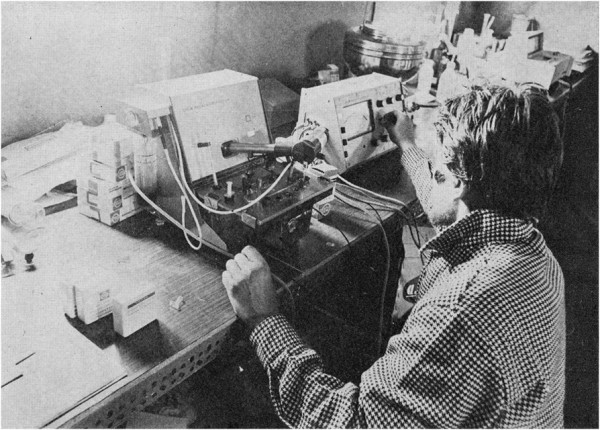
The author recording blood gas partial pressures by microelectrodes.

My main task in the expedition was to develop my research project in the facility provided by the organization. I was assisted by a technician from my laboratory and, occasionally, by one of the three doctors participating in the expedition with professional assignments. A large number of porters and altitude Sherpas (70) were also recruited. I had obtained informed consent from all Italian members and a number of selected altitude Sherpas to collaborate as subjects for some of the experiments that were planned. The equipment was provided by the organization and later donated to my laboratory at the University of Milano. It included a special custom-built metabograph conceived for measurements of maximum oxygen consumption in a closed circuit opposing low resistance up to very high ventilation rates, breathing ambient air or pure oxygen [11], CO_2_ and O_2_ breath-by-breath meters, an earpiece oximeter coupled to amplifiers and a recorder, battery-powered electrocardiographs, PO_2_, PCO_2_, and pH microelectrodes, a spectrophotometer, and the basic equipment for a standard laboratory of clinical biochemistry (see pictures). The laboratory was moderately heated (Figures 4,5,6).

Several variables were recorded in selected groups of Caucasians, before and after 8 weeks of continuous (5,000–7,000 m) or intermittent (2–6 h daily, between 2,800 and 7,000 m) altitude exposure, and in Sherpas operating at or above the base camp. Among these were RBC, [Hb], and hematocrit (Hct); *V*′O_2_max, *V*′Emax, HRmax, and %SatHbO_2_; resting and exercise cardiac output, *Q*′; *V*′O_2_max changes when administering hypobaric oxygen or normoxic air–oxygen mixtures to breathe to acclimatized Caucasians with increased Hct at the Mt. Everest base camp [12]; and peak blood lactate concentration at exhaustion ([La_b_]_p_) and the kinetics of payment of the lactacid oxygen debt [13].

The results of *hematologic measurements* confirmed essentially previous data obtained in similar conditions. An original finding concerning blood composition appears in Tables 1 and 2: it confirms the different responses to chronic hypoxia of native Sherpas vs. acclimatized Caucasians, also showing an unexpected, peculiar reaction of the members of the helicopter crew.

**Table 1:** **Hematologic data from different groups of subjects (*n *= 13): Caucasian mountaineers (altitude vs. sea level)**

	**Age (years)**	**Weight (kg)**	**RBC (cells/mm**^**3**^**)**	**Hb (g%)**	**HRmax (bpm)**	***V'*O**_**2**_**max (indirect method)**	
						**l/min**	**ml/kg/min**
Sea level	29.1 ± 5.9 (S.D.)	71.5 ± 9.7	4.70 ± 0.22	14.96 ± 0.86	187 ± 13	3.13± 0.29	44.30 ± 5.58
Altitude base camp (5.350 m)		66.9 ± 8.7	6.65 ± 0.7	21.60 ± 2.42	160 ± 9	2.36 ± 0.29	35.73 ± 6.20
Altitude Lukla (2,850 m)		66.9 ± 8.7	6.65 ± 0.7	21.60 ± 2.42	168 ± 15	3.03 ± 0.25	45.77 ± 5.28

**Table 2:** Caucasians (two groups) vs. Sherpas

**Subjects**	**Number**	**Exposure**		** RBC (cells/mm**^**3**^**)**	**Hb (g%)**	**Hct (%)**
		**Type**	**Time (weeks)**			
Italian (mountaineers)	36	Continuous	8	7.03 ± 0.66 (S.D.)	23.52 ± 1.64	66.34 ± 4.31
Sherpas	21	Continuous	8	5.02 ± 0.80	20.10 ± 1.26	62.66 ± 4.69
Italians (helicopter personnel)	11	Intermittent (2–6 h daily up to 6,300 m)	5	5.02 ± 0.45	19.6 ± 1.32	55.5 ± 2.40

The latter, based at 2,850 m, were regularly making use of supplemental oxygen when operating at above 4,000 m and used to spend several hours a day on the ground in the altitude range of 4,500–6,300 m. They were apparently very well acclimatized from the respiratory standpoint, as proven by the perfect altitude tolerance when stranded at above 6,300 m, but were characterized by hematologic parameters similar to those of Sherpas. To the author's knowledge, these measurements have never been repeated.

**Figure 7:**
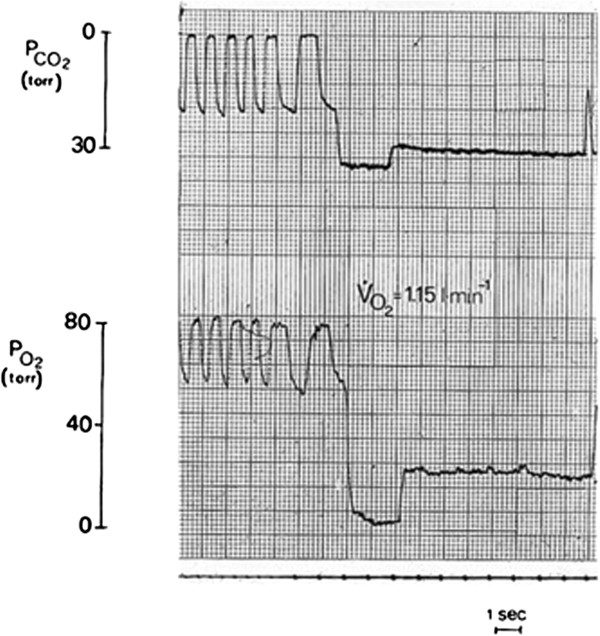
**Mt. Everest base camp: rebreathing CO**_**2**_**(*top*) and O**_**2**_**(*bottom*) plateaux.** Obtained after inhalation of a 3-l *V*_T _of a 8% CO_2_-N_2 _mixture during a moderate cycling exercise. Rapid-responding O_2 _and CO_2 _analyzers were used.

**Figure 8:**
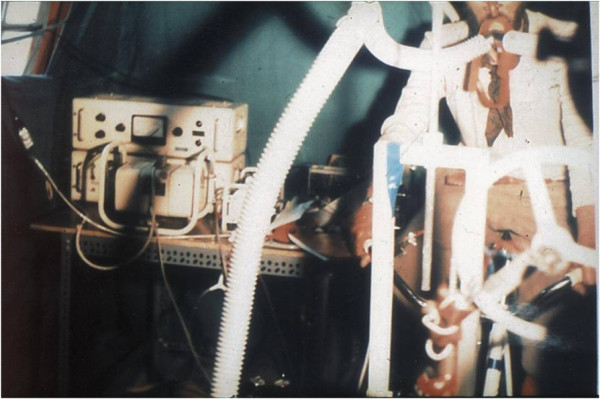
**Mixed venous**$P_{O_{2}}$**and**$P_{{\mathrm{CO}}_{2}}$**being recorded on Rinaldo Carrel at the base camp after descent from the summit.**

**Figure 9:**
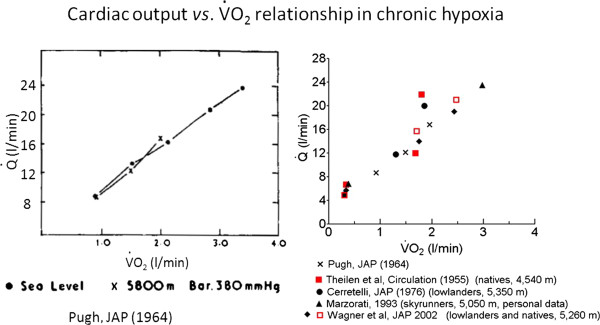
***Q*′ vs. *V*′O**_**2 **_**relationship in chronic hypoxia. **By Pugh (1964) (*left*) and by the author at Mt. Everest and, later, by other investigators (*right*).

Measurements of *cardiac output* by the nitrogen–carbon dioxide rebreathing method (Figures 7 and 8) [14] and of O_2_ consumption have been carried out at the base camp (5,350 m) in five subjects at rest and, in two of them, at increasing exercise loads on the bicycle ergometer and compared with the sea level control values (Figure 9) [15]. Resting *Q*′ values were found to be unchanged as were the measurements obtained at moderate workloads. At about 90% of their *V*′O_2_max, the two investigated subjects attained *Q*′ levels of 19.0 and 20.7 l/min, respectively, vs. sea level control data of 20.9 and 23.7 l/min. *V*′O_2_max-extrapolated *Q*′max values were 10% lower than the control values at sea level while maximum heart rates were 148 and 162 beats/min, respectively (vs. 160 and 178 at sea level). Thus, the stroke volume of the heart in the prevailing experimental conditions was essentially unchanged.

**Figure 10:**
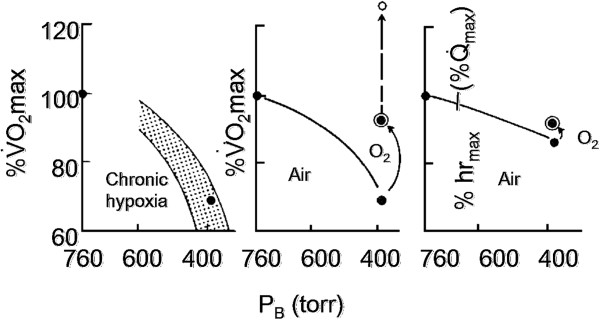
**The effect of acute normoxia on *V*′O**_**2**_**max at the Mt. Everest base camp.**

A major interest for me was the analysis of the *factors limiting maximal exercise at altitude* in acclimatized lowlanders. By the use of the metabograph, it was possible to show, right at the base camp (Pb = 410 Torr), that these subjects (*n* = 10), when given 100% oxygen to breathe, could raise their maximum oxygen uptake from an average of 70% of the control sea level value only to 92% (Figure 10) [15]. Considering the limited reduction of maximum cardiac output and the 40% increase in blood Hb concentration, to explain these results (also confirmed by indirect measurements of *V*′O_2_max in a group of 13 subjects taken down by the helicopter to Lukla, 2,850 m), two hypotheses were put forward, i.e., (a) that the release of oxygen in the muscles in peripheral capillaries could have been impaired by packing of erythrocytes due to extremely high Hct and/or (b) that the blood was shunted away from the muscles to reduce the load on the heart due to increased viscosity. Neither condition has been tested experimentally. However, more recent findings (see the next paragraph) indicate that the reduced maximum rate of oxidations could have been mainly the result of muscle deterioration (reduction of tissue mass, decreased mitochondrial volume density, increase in the accumulation of lipofuscin, a marker of damage by reactive oxygen species, and enzymatic impairments of the muscle metabolic machinery).

**Figure 11:**
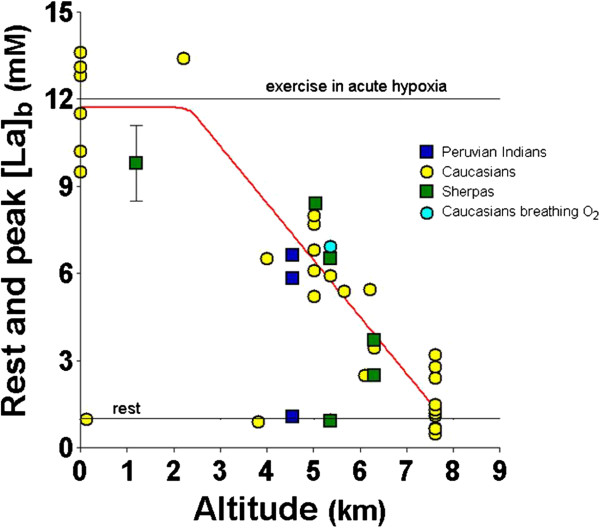
**Rest and [La**_**b**_**]**_**p**_**. **As a function of altitude in various groups of subjects as indicated in the enclosure.

In the course of the expedition, I had the opportunity to evaluate in a large group of subjects, both acclimatized Caucasians and Sherpas, and in various experimental conditions the apparent peak contribution to energy metabolism by anaerobic glycolysis. The Sherpas, for most of the time, were living at altitudes varying between 2,500 and 3,400 m, whereas during the expedition, they spent, like Caucasians, at least 8 weeks between 5,000 and 7,500 m. Figure 11 summarizes the results.

Both breathing ambient air or O_2_, resting lactate concentrations at altitude are approximately the same as those at sea level. By contrast, [La_b_]_p_ at exhaustion (work duration 3–5 min) appears to be considerably lower than at sea level in all investigated groups. These data were among the first after those of Dill et al. [16] and Edwards [17] confirming the existence of an apparent blunting of the maximum energy yield from anaerobic glycolysis at altitude. The latter phenomenon was later defined the ‘lactate paradox’ by Hochachka, and several studies were carried out on this topic at the Pyramid laboratory and upon return from various thematic research missions along the Khumbu Valley in Nepal by my collaborators, particularly on the quantitative aspects and the time course of the process, both during acclimatization and deacclimatization [18]. The quantitative role of anaerobic glycolysis in acclimatized Caucasians after supramaximal exercise was also determined [19]. For over three decades, several explanations, mostly unsatisfactory, of this finding were attempted. Only recently, after carrying out joint muscle and blood analyses on acclimatized Caucasians and Bolivian natives, Van Hall et al. [20] came to the conclusion that a lactate paradox ‘does not exist,’ in the sense that the maximum potential of anaerobic glycolysis in exercising muscles in hypoxia is not reduced. Thus, the innumerable data from most blood studies underlying the so-called lactate paradox in chronic hypoxia are likely attributable to the status of the lactate and hydrogen ion transporters which could affect the kinetics of lactic acid equilibration between the blood and muscle tissue. In retrospect, some hints that the lactate paradox was not the expression of an impairment of anaerobic glycolysis could have been obtained by a more critical analysis of the curve of lactate disappearance from the blood after exhaustive exercise at the Mt. Everest base camp, both when breathing ambient air or oxygen. Indeed, at altitude, at variance with sea level measurements, long delays (over 20 min) were observed [13] before lactate concentration started dropping prior to resuming the well-known monoexponential function characterized by a half time of approximately 15 min, both at sea level and at altitude.

### Morphological and functional adaptation of human skeletal muscle to chronic hypoxia

**Figure 12:**
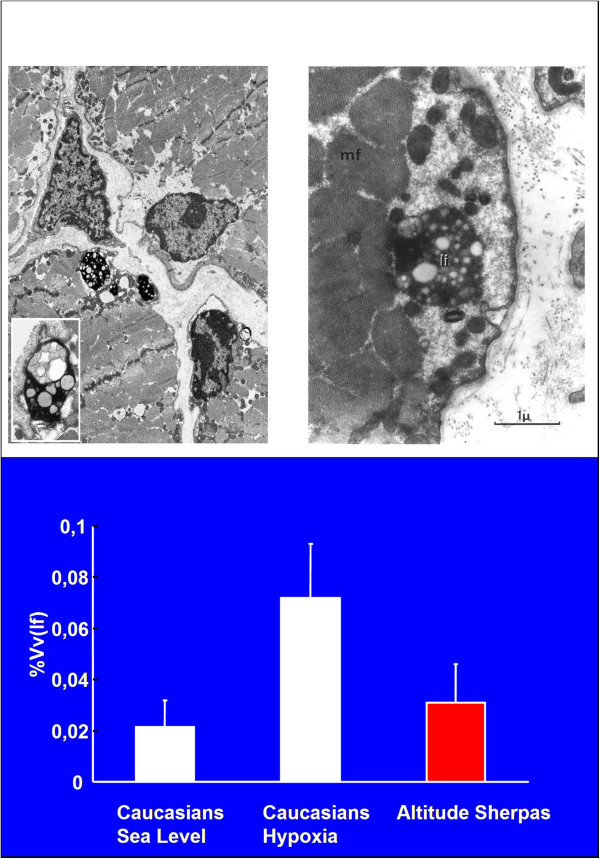
**Accumulation of lipofuscin and quantitative changes. **Accumulation of lipofuscin in the muscle (vastus lateralis) of a Caucasian mountaineer and a Sherpa. Quantitative changes appear in the histogram.

This work was conceived during my stay in Geneva and was carried out in collaboration with Hans Hoppeler from the Department of Anatomy of the University of Bern (Switzerland) and Hans Howald from the Swiss School for Physical Education and Sports of Magglingen (Switzerland) on members of the Swiss expeditions of 1981 to Mt. Lothse-Shar (8,398 m) and of 1986 to Mt. Everest (8,848 m). Muscle macro- and microscopic structural changes were assessed in two groups of mountaineers from CT scans of the thigh and from muscle biopsies of the vastus lateralis muscle, respectively, before and after return to Switzerland following their 8–10 weeks of sojourn at altitudes ranging from 5,000 and 8,850 m [21-23]. Together with an approximately 15% reduction of muscle mass, signs of tissue deterioration were observed consisting of accumulation of lipofuscin as evidence of fiber catabolism in subsarcolemmal locations (Figure 12).

The reduction of muscle mass was accompanied by a 20%–25% decrease of mitochondrial volume density assessed by electron microscopy and by a proportional drop of oxidative enzyme activity as shown by photometric analyses in bioptic samples from the vastus lateralis, whereas only moderate changes in glycolytic enzyme activity were observed [24].

### Research at the Pyramid

**Figure 13:**
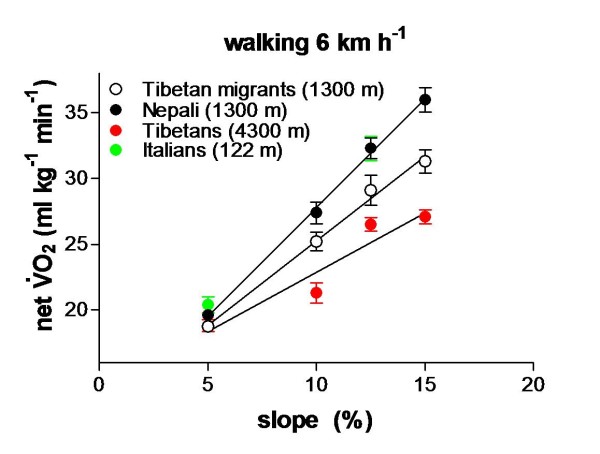
**Better walking economy in altitude natives. **net *V*′O_2_ during walking at increasing grades at a constant speed of 6 km/h is lower in Tibetan natives exercising at altitude.

The interest of my group over the final decade of the last century was mainly centered on the study of the factors affecting *V*′O_2_max at altitude. In the course of several missions to the Khumbu Valley and the Pyramid, we investigated the main metabolic, respiratory, and cardiovascular responses to peak exercise of groups of subjects characterized by different ethnic backgrounds, altitude exposure histories, and training conditions during a standardized (approximately 4 weeks) sojourn at 5,050 m. To my knowledge, this is still the only comparative study carried out at this altitude on homogeneous groups of selected subjects in identical environmental conditions, using the same protocols and experimental setup, thereby reducing to a minimum possible confounding factors. Two novel findings were reported: The first is that second-generation Tibetan lowlanders compared to untrained (UT) and trained (T) Caucasians after the sojourn of approximately 30 days at the Pyramid were able to develop, on average, 92% *V*′O_2_ peak of their pre-exposure (approximately 1,300 m), whereas UT and T Caucasians were at 70% and 55%, respectively. The second is that, within both the Tibetan and Caucasian groups, subjects characterized by higher maximum oxidative power at low altitude did lose a greater fraction of their control value at 5,050 m [25]. Another interesting finding is that the superior work capacity of high-altitude natives is not only based on relatively small positive differences in specific *V*′O_2_ peak data at altitude but also, particularly, on a more efficient economy level as determined in cycling, walking, and running on the treadmill [26,27]: The latter result is probably due to metabolic adaptations such as increased muscle myoglobin content, better antioxidant defense, and increased efficiency of oxidative phosphorylation (Figure 13).

### Revisiting energy metabolism at altitude on the basis of recent findings of molecular physiology

**Figure 14:**
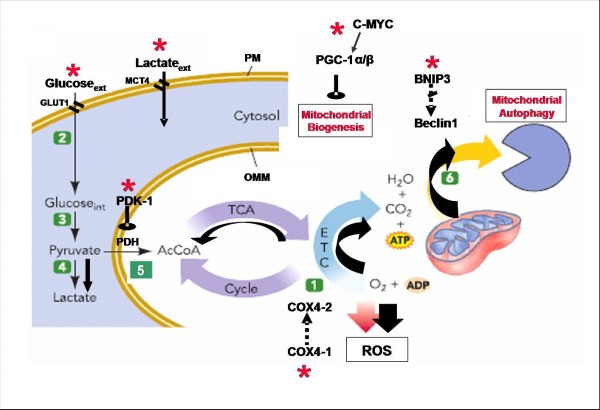
**Scheme of the regulation of energy metabolism in hypoxia.***Red asterisks *appear to be putative sites of control, by HIF-1α, of membrane transport of glucose and/or lactic acid, enzymatic reactions along the glycolytic chain, the tricarboxylic acid cycle, the oxidative phosphorylation sequence, and mitochondrial biogenesis and autophagy. *Beclin 1* is an essential autophagy protein; *BNIP 3*, a pro-apoptotic mitochondrial protein; *c-Myc*, target genes involved in cell growth apoptosis, etc.; *COX*, complex IV subunits 1, 2; *PDK-1*, PDH kinase; *ETC*, electron transport chain; *Glut 1*, glucose transporter 1; *MCT4*, plasma membrane lactate transporter 4; *OMM*, outer mitochondrial membrane; *PGC-1α/1β*, key regulators of energy metabolism; *ROS*, reactive oxygen species; and *TCA*, tricarboxylic acid cycle.

Over the last few decades, most studies on humans exposed to hypobaric hypoxia have been aimed at assessing the functional adjustments assuring homeostasis of oxygen delivery to tissues. Data on blood composition, oxygen equilibrium curve, respiratory and cardiovascular variables, and aerobic and anaerobic energy metabolism at rest and at exercise appear to be affected by a large distribution that cannot be explained by inter-individual variability and/or by differences in measuring procedures alone. The recent appearance of a new player, the hypoxia-inducible factor-1 (HIF-1) which is the master regulator of cell hypoxic signaling and of genes whose products play a large number of metabolic functions, opens a new scenario for an updated interpretation of several earlier results (Figure 14) A holistic approach is being attempted whereby the role of proteins known to act on isolated cells are being identified and quantitated also in man [28,29].

The study of muscle energetics recently made a substantial step forward, thanks to the development of advanced proteomic techniques. In particular, the use of differential proteomics and the adoption of stringent statistical methods have made it possible to identify a large number of qualitative and quantitative protein changes induced by altitude exposure. These data will allow us to unravel, among others, the complex metabolic adaptation process of muscle to hypobaric hypoxia. In this context, together with C. Gelfi, I investigated by 2DE and ESI MS/MS on approximately 500 protein spots the differences in the vastus lateralis muscle contractile and metabolic enzymatic protein pattern among altitude Tibetan natives, second-generation Tibetans born in Kathmandu, and Nepali lowlanders of Indian ethnicity. The analysis allowed us to identify some of the mechanisms by which altitude populations seem to adapt metabolic control and prevent muscle damage from hypoxia thereby improving physical activity under extreme conditions [30]. Another interesting study [31] dealt with adaptive muscle protein modulation in the early phase (7–9 days) of exposure to hypobaric hypoxia at the Capanna Margherita (4,559 m). The results showed that a large number of proteins involved in iron transport, tricarboxylic acid cycle, oxidative phosphorylation, and response to oxidative stress were significantly decreased. By contrast, at the end of the sojourn, HIF-1α and pyruvate dehydrogenase kinase were at the pre-hypoxia levels indicating that the muscle early adaptive response to hypoxia does not involve the stabilization of the master regulator of hypoxic signaling. At present, I am involved in a systematic investigation, in selected sea-level Caucasian participants in the CXE, of the functional adaptation to hypoxia of metabolic, stress response, and contractile muscle proteins as a function of altitude and exposure duration up to 66 days.

## Personal contributions to extreme physiology besides altitude

My interest in extreme physiology and physiopathology offered me, over the years, the opportunity to undertake research on different topics besides altitude hypoxia, both on healthy subjects and patients:

1. *Top athletes*. In 1960, in a laboratory installed within the Olympic Village during the Olympic Games of Rome, together with Margaria and several colleagues, I had the opportunity to determine several physiological variables, particularly *V*′O_2_max, in 40 top athletes, most of them medalists, practicing different track and field specialties, swimming, and rowing [32]. I had also the chance to examine some of the best road bicycle runners, winners of the Tour de France and of the Giro d'Italia, including Eddy Merkx, and some top cross country skiers. Among others, these data appear nowadays particularly interesting for comparisons with homologous athletes.

2. *Highland populations*. Within the International Biological Program (1961), I participated in the highland of Kenya in the measurement of the maximum aerobic and anaerobic power of large groups of normal Drobo Masai as a function of age and gender and to compare them with other populations [33].

3. *Extreme divers*. In the open sea of Sicily and, later, in the ‘wet’ hyperbaric chamber of the SUNY at Buffalo, I participated with Claes Lundgren, David Pendergast, Guido Ferretti, and Massimo Ferrigno in the assessment of cardiovascular, respiratory, and metabolic variables in the course of extreme simulated dives by the three members of the Majorca family [34].

4. *Astronauts*. During the Columbia STS 78 LMS mission (16 days), I was involved in a study of muscle fatigue in microgravity and in the following recovery phase [35] which was preliminary to the ISS Sarcolab project on neuromuscular adaptation during a 6-month flight that is taking place at the present time and in which I am involved with a study of muscle deterioration assessed by proteomic techniques.

Concerning *pathophysiology and medicine*, a major personal contribution during my assignment in Geneva was the organization of an international study [36] carried out in collaboration among the Exercise Laboratory of the Consiglio Nazionale delle Ricerche of Milano (Dr. C. Marconi), the University of Paris Medical School (Prof. M. Rieu and Prof. C. Cabrol), and the Max Planck Institut für Experimentelle Medizin at Göttingen (Dr. M. Meyer) on the characteristics of aerobic fitness and its determinants in *heart transplant recipients* inevitably affected by heart denervation [37]. The experiments were aimed at evaluating the specific role of cardiac, pulmonary, and muscle factors in limiting the performance of these patients, particularly those (children) showing signs of heart reinnervation. The denervated heart model has proven to be suitable for discriminating between cardiovascular and metabolic limitations and for assessing the benefits from current rehabilitation procedures. During one of the experimental sessions, we had the probably unique opportunity to determine the pulmonary gas exchange kinetics on a lady, recipient of a Jarvik-7 total artificial heart, whom I do not hesitate to consider heroic [38].

## Conclusion

It is fair to acknowledge that starting from the turn of the millennium, my activity has dealt much more with project planning than with work in the laboratory. The names of most colleagues participating in the studies appear in the reference list of this paper. I apologize to those that might have been omitted due to the failure of my dim memory. To my extreme satisfaction, most of them have surpassed their master's degree and are now teaching and also carrying out research in fields connected with extreme physiology and medicine in Italy, Switzerland, the UK, the USA, and Africa. They make up, distributed in several areas, the fourth generation of the Angelo Mosso followers.

And last but not the least, I would like to acknowledge that I enjoyed my career and if I had to do it all over again, I would: Indeed, it was made possible by passionate work but mainly by a fortunate combination of circumstances, by the support of my parents and, particularly, of my wife (Maria Grazia, a psychologist) and of my two daughters (Silvia, an architect and Claudia, a high school teacher of French literature and, soon, a Jungian psychoanalyst). However, I must admit that the profession of Maria Grazia and the youth of Silvia and Claudia were severely influenced by the burden imposed on them by the innumerable changes of places, customs, languages, schools, friends, and the distance of most family members. For all this, I feel fully responsible and, unfortunately, unforgivable. Luckily, my wife can still bear me, whereas Silvia and Claudia have happy families in Geneva.

### Photographs of the author

**Figure 15:**
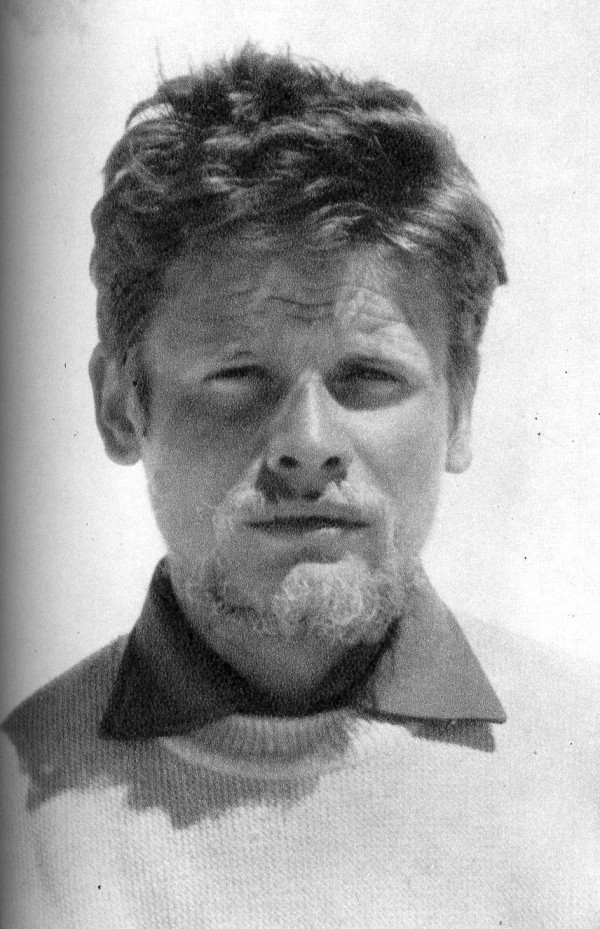
At Mt. Kanjut-Sar (1959).

**Figure 16:**
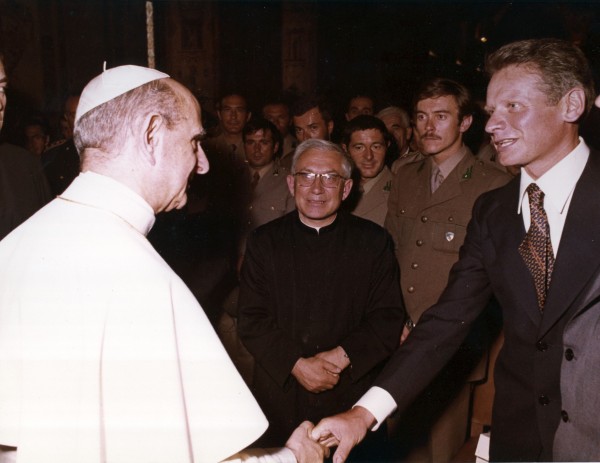
Visiting Pope Paul VI upon returning from the Mt. Everest Expedition (1973).

**Figure 17:**
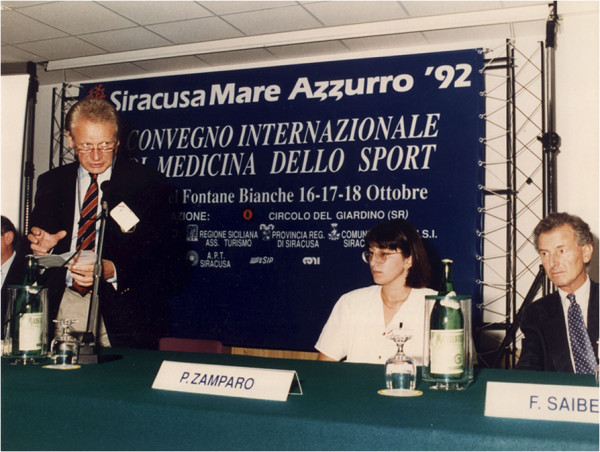
At a meeting in Siracusa (Sicily) (1992).

**Figure 18:**
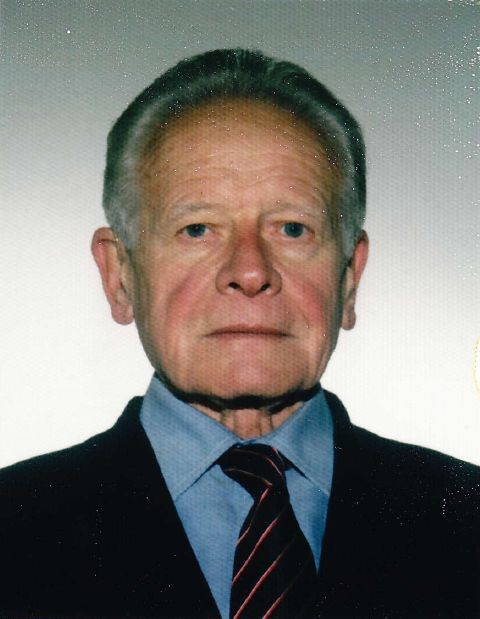
Recent picture (2013).

My physical ‘deterioration’ as a function of age is documented by the following four pictures (Figures 15,16,17,18).

## Abbreviations

[Hb]: Hemoglobin concentration; Hct: Hematocrit; HIF-1: Hypoxia-inducible factor-1; [Lab]p: Peak blood lactate concentration; PAO2: Alveolar O_2 _partial pressure; Q′: Cardiac output; V′O2max: Maximum oxygen consumption; T: Trained; UT: Untrained.

## Competing interests

The author declares that he has no competing interests.
